# Eye Movement Abnormalities in Major Depressive Disorder

**DOI:** 10.3389/fpsyt.2021.673443

**Published:** 2021-08-10

**Authors:** Junichi Takahashi, Yoji Hirano, Kenichiro Miura, Kentaro Morita, Michiko Fujimoto, Hidenaga Yamamori, Yuka Yasuda, Noriko Kudo, Emiko Shishido, Kosuke Okazaki, Tomoko Shiino, Tomohiro Nakao, Kiyoto Kasai, Ryota Hashimoto, Toshiaki Onitsuka

**Affiliations:** ^1^Department of Neuropsychiatry, Graduate School of Medical Sciences, Kyushu University, Fukuoka, Japan; ^2^Institute of Industrial Science, The University of Tokyo, Tokyo, Japan; ^3^Department of Pathology of Mental Diseases, National Institute of Mental Health, National Center of Neurology and Psychiatry, Kodaira, Japan; ^4^Department of Rehabilitation, The University of Tokyo Hospital, Tokyo, Japan; ^5^Department of Neuropsychiatry, Graduate School of Medicine, The University of Tokyo, Tokyo, Japan; ^6^Department of Psychiatry, Graduate School of Medicine, Osaka University, Suita, Japan; ^7^Japan Community Health Care Organization Osaka Hospital, Osaka, Japan; ^8^Life Grow Brilliant Mental Clinic, Osaka, Japan; ^9^Graduate School of Science and Technology, Nara Institute of Science and Technology, Ikoma, Japan; ^10^Division of Cerebral Integration, Department of System Neuroscience, National Institute for Physiological Sciences, Okazaki, Japan; ^11^Department of Psychiatry, Nara Medical University, Kashihara, Japan; ^12^Division of Developmental Emotional Intelligence, Research Center for Child Mental Development, University of Fukui, Fukui, Japan; ^13^United Graduate School of Child Development, Osaka University, Kanazawa University, Hamamatsu University School of Medicine, Chiba University and University of Fukui, Suita, Japan; ^14^The International Research Center for Neurointelligence (WPI-IRCN), The University of Tokyo Institutes for Advanced Study, Tokyo, Japan

**Keywords:** major depressive disorder, free-viewing test, fixation stability test and smooth pursuit test, alerted aging effect, discriminant analysis, eye movements

## Abstract

**Background:** Despite their high lifetime prevalence, major depressive disorder (MDD) is often difficult to diagnose, and there is a need for useful biomarkers for the diagnosis of MDD. Eye movements are considered a non-invasive potential biomarker for the diagnosis of psychiatric disorders such as schizophrenia. However, eye movement deficits in MDD remain unclear. Thus, we evaluated detailed eye movement measurements to validate its usefulness as a biomarker in MDD.

**Methods:** Eye movements were recorded from 37 patients with MDD and 400 healthy controls (HCs) using the same system at five University hospitals. We administered free-viewing, fixation stability, and smooth pursuit tests, and obtained 35 eye movement measurements. We performed analyses of covariance with group as an independent variable and age as a covariate. In 4 out of 35 measurements with significant group-by-age interactions, we evaluated aging effects. Discriminant analysis and receiver operating characteristic (ROC) analysis were conducted.

**Results:** In the free-viewing test, scanpath length was significantly shorter in MDD (*p* = 4.2 × 10^−3^). In the smooth pursuit test, duration of saccades was significantly shorter and peak saccade velocity was significantly lower in MDD (*p* = 3.7 × 10^−3^, *p* = 3.9 × 10^−3^, respectively). In the fixation stability test, there were no significant group differences. There were significant group differences in the older cohort, but not in the younger cohort, for the number of fixations, duration of fixation, number of saccades, and fixation density in the free-viewing test. A discriminant analysis using scanpath length in the free-viewing test and peak saccade velocity in the smooth pursuit showed MDD could be distinguished from HCs with 72.1% accuracy. In the ROC analysis, the area under the curve was 0.76 (standard error = 0.05, *p* = 1.2 × 10^−7^, 95% confidence interval = 0.67–0.85).

**Conclusion:** These results suggest that detailed eye movement tests can assist in differentiating MDD from HCs, especially in older subjects.

## Introduction

Major depressive disorder (MDD) is a common global disorder that affects over 264 million people (1). Depression is ranked as the single largest contributor to global disability (7.5% of all years lived with a disability) (2) and has been one of the top three leading causes of health loss for nearly three decades (3). The lifetime prevalence of MDD was reported to be 14–17% with a 1-year prevalence of 4–8% (WHO, 2020). Many studies have attempted to elucidate the pathophysiology of depression; however, it remains poorly understood. Stressful events, genetic vulnerability, environmental interactions, abnormalities in several neurotransmitters, inflammation, as well as alterations in neuropeptides and hormones have been investigated as causes of MDD (4–6). Natural disasters (7), pandemics such as COVID-19 (8–10), and cultural differences (11–14) also have a huge impact on the development of MDD.

Clinically, it is difficult to diagnose mood disorders in patients with MDD and bipolar disorder (15). For example, during the initial evaluation, patients with bipolar disorder sometimes show only depressive symptoms and thus receive antidepressants based on a diagnosis of MDD, which may cause several critical problems (16, 17). For this reason, objective indices for MDD are needed; however, to date, none have been established.

Eye movements are considered a potential biomarker for the diagnosis of mental illness (18, 19). We previously showed an integrated score using three measurements (scanpath length during a free-viewing test, horizontal position gain during the fast Lissajous paradigm in a smooth pursuit test, and the duration of fixations during the far distractor paradigm of a fixation stability test) could distinguish between patients with schizophrenia (SZ) and HCs with 82% accuracy (20). A recent review has provided convincing evidence of eye-movement abnormalities in SZ (21, 22). However, Smyrnis et al. (23) noted that the sensitivity of eye movement deficits to differentiate psychiatric patients from healthy controls (HCs) was not high enough to be clinically relevant for diagnostic purposes. In the previous study (20), our group reported that eye movement can be a useful biomarker for schizophrenia. In order to use eye movements as a diagnostic tool, it is necessary to discriminate schizophrenia from other psychiatric disorders. Therefore, we conducted the same tasks used in the previous studies.

As listed in Table 1, several studies have used different methods to evaluate eye movements in MDD. Iacono et al. (24) reported that the performance of the MDD group was not significantly different from that of the HC group regarding smooth-pursuit eye movement (SPEM), but smooth-pursuit tracing errors were greater for those with a higher frequency of episodes of the disorder. Abel et al. (25) studied smooth pursuit gain and catch-up saccade (CUS) in affective disorders and found that when the constant stimulus velocity was 5°/s, but not 20°/s, MDD patients had higher CUS rates than HCs. Malaspina et al. (26) studied the effects of electroconvulsive therapy (ECT) on SPEM with severe MDD and reported that SPEM was transiently disrupted but pursuit performances were improved after two sessions of ECT and at 2 months follow-up. They concluded that SPEM abnormalities may be a state marker in severe MDD. Flechtner et al. (27) explored SPEM in thirty-four MDD patients and found they exhibited lower pursuit gain and higher CUS rates than HCs. Flechtner et al. (28) also reported that SPEM performance was not influenced by medication or clinical state in a test-retest study. Fabisch et al. (29) reported no significant difference between unipolar depressed patients and HCs. Li et al. (31) reported that compared with the HC group, the MDD group had a significantly shorter duration and more saccades in a fixation stability test. Taken together, these studies indicate inconsistent findings regarding eye movement abnormalities in MDD.

**Table 1:** Demographic and clinical characteristics of the study subjects and major findings of patients with MDD in eight eye movement studies.

		**MDD patients**							**Healthy controls**						
			**Age (years)**		**Male**		**Currently on medication**		**Age (years)**			**Male**			
**Study**	**Task**	***N***	**Mean**	**SD**	***N***	**%**	***N***	**%**	***N***	**Mean**	**SD**	***N***	**%**	**Parameters**	**Major findings in MDD**
Iacono et al. (24)^a^	SPEM	25	37.9	12.9	5	20	N/A	N/A	46	35.0	11.9	8	22.9	RT, RMSE	All variables: no significance
Abel et al. (25)^b^	SPEM	16	48.4	12.4	16	100	4	25	21	37.5	10.9	21	100	TWAG, CUS rates, CUS amplitude	Higher CUS rates in 5°/s SPEM
Malaspina et al. (26)	SPEM	18	28.9	5.6	N/A	N/A	0	0	20	30.6	7	N/A	N/A	% abn, Large saccades	All variables: no significance
Flechtner et al. (27)	SPEM	34	46.9	11	9	26.5	30	88.2	42	34.3	10.9	20	47.6	Pursuit gain, CUS, Anticipatory saccade, BUS, SWJ	Lower pursuit gain
Flechtner et al. (28)^c^	SPEM	34	46.9	11	9	26.5	34	100	42	34.3	10.9	20	47.6	Pursuit gain, CUS, Anticipatory saccade, BUS, SWJ	All variables: no significant difference between all time
Fabisch et al. (29)	SPEM	19	34.4	8.3	19	100	19	100	21	37.8	5.9	21	100	Peak gain, CUS error, CUS velocities	All variables: no significance
Chen et al. (30)	FVT	19	28.3	4.7	N/A	N/A	0	0	19	27.9	4.6	N/A	N/A	NF, tFD, aFD	More NF, longer tFD and aFD
Li et al. (31)	FST	60	25.4	7.2	N/A	N/A	0	0	60	24.2	6.1	N/A	N/A	NF, FD, Number of saccades, Saccade path	More NF and number of saccades, shorter FD, longer saccade path
	FVT													NF, Duration of saccade, Saccade amplitude, mFD, Saccade path	Shorter duration of saccade, longer mFD

*SPEM, Smooth pursuit eye movement; FVT, Free-viewing test; FST, Fixation stability test; RT, Reaction time; RMSE, Root mean square error; TWAG, Time-weight average gain; CUS, Catch-up saccade; BUS, Back-up saccade; SWJ, Square wave jerk, NF, number of fixations; tFD, total fixation duration; aFD, average fixation duration; FD, fixation duration; mFD, mean fixation duration*.

a *This study analyzed remitted MDD*.

b *This study analyzed affective disorders (MDD non-psychotic = 10, MDD psychotic = 1, bipolar depression non-psychotic = 4, schizoaffective disorder = 1)*.

c *A longitudinal study by Flechtner et al. (27)*.

In the present study, we recorded eye movements and determined the detailed characteristics of eye movements in MDD patients at multiple facilities. We then examined how eye movements might be useful for differentiating between MDD and HCs.

## Materials and Methods

### Subjects

Patients with MDD recruited from Kyushu University Hospital, Osaka University Hospital, The University of Tokyo Hospital, and Nagoya University Hospital were diagnosed by two or more trained psychiatrists according to criteria from the DSM-IV based on the Structured Clinical Interview for DSM-IV (SCID). All subjects were biologically unrelated, were of Japanese descent, and had no history of ophthalmologic disease, or neurological/ medical conditions that might influence the central nervous system. Specific exclusion criteria included atypical headaches, head trauma with loss of consciousness, thyroid disease, epilepsy, seizures, substance-related disorders, or intellectual disability. HCs were recruited through regional advertisements and were evaluated for psychiatric, medical, and neurological concerns using the non-patient version of the SCID to exclude individuals with current or past contact with psychiatric services or who had received psychiatric medication. Eye movements were recorded from 51 patients with MDD and 519 HCs, who were recruited from Kyushu University (28 MDD, 29 HCs), Osaka University (15 MDD, 333 HCs), The University of Tokyo (6 MDD, 70 HCs), Nagoya University (2 MDD, 48 HCs), and Nara Medical University (0 MDD, 40 HCs). Each facility used a common protocol and analysis manual, and quality control was performed every 2 months to ensure uniformity of the data. We used the data from 37 patients with MDD and 400 HCs, for which the quality of the data was ensured by rigorous quality checks. Current symptoms of depression were evaluated using the Hamilton Depression Scale (HAM-D) (32) and the total dosages of prescribed the antidepressant benzodiazepine or antipsychotics were calculated using imipramine (IMP), diazepam (DZP), and chlorpromazine (CPZ) equivalents (mg/day) (33). The demographic information of the study subjects is shown in Table 2. Based on the criteria for depression (34), 15 patients showed mild depression, 9 showed moderate depression, 1 showed severe depression and 9 were euthymic. HAM-D scores of the three patients were unknown.

**Table 2:** Demographics of the HC and MDD groups.

	**HCs**	**MDD**	***χ^2^* or *t***	***p*-value**
Male/female	201/199	18/19	0.04	0.85
Age (years)	35.3 ± 14.8	49.5 ± 11.6	−7.0	8.6 × 10^−9^^*^
Education (years)	15.4 ± 2.3	14.5 ± 2.4	2.1	0.41
Premorbid IQ	107.7 ± 8.1	106.9 ± 10.9	0.38	0.71
Current IQ	107.1 ± 12.0	100.4 ± 13.5	2.7	6.7 × 10^−3^^*^
Onset age (years)		36.9 ± 13.6		
Duration of illness (years)		13.0 ± 8.4		
HAM-D		12.0 ± 6.3		
IMP (mg)		187.9 ± 150.4		
DZP (mg)		6.0 ± 5.9		
CPZ (mg)		61.9 ± 117.1		

*Premorbid IQ was estimated using the Japanese Adult Reading Test*.

*Current IQ was estimated using the Wechsler Intelligence Scale short form*.

*HAM-D, Hamilton Depression Rating Scale; IMP, imipramine; DZP, diazepam; CPZ, chlorpromazine*.

* *p < 0.05*.

The study was performed in accordance with the World Medical Association's Declaration of Helsinki and was approved by the Research Ethical Committees of Kyushu University, Osaka University, The University of Tokyo, Nagoya University, and Nara Medical University. All participants provided written consent to the study after a full explanation of the study procedures. Anonymity was preserved for all participants.

### Eye Movement Recordings and Processing of Eye Movement Data

The subjects faced a 19-inch liquid crystal display monitor placed 70 cm from the observers' eyes. Visual stimuli were presented using MATLAB (The Mathworks, Natick, MA, USA) *via* the Psychophysics Toolbox extension (35). The eye movements and pupil areas of the left eye were measured at 1 kHz using the EyeLink1000 Plus (SR Research, Ontario, Canada) system.

Eye position data were smoothed with a digital FIR filter (−3 dB at 30 Hz), and the eye velocity and acceleration traces were obtained using a two-point forward difference algorithm to identify saccadic eye movements. Eye movement records were segmented into the blink, the saccade, and the fixation periods. Further details are described in Supplementary Methods.

### Eye Movement Paradigms and Extracted Measurements

**Figure 1:**
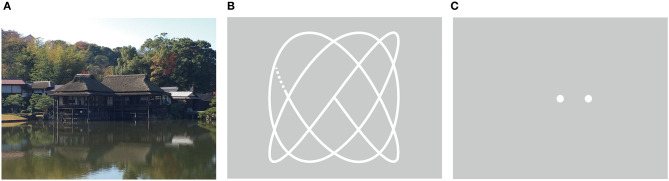
Eye movement paradigms. **(A)** Free-viewing test*. **(B)** Lissajous trajectories of the smooth pursuit eye movement test. **(C)** Fixation stability test. The fixed target (center) and a distracter stimulus (right). *This image example is a photo taken by the author which is not the actual one used for the test.

We administered 3 eye movement paradigms (free-viewing test, fixation stability test, and smooth pursuit test) and obtained 35 eye movement measurements comprising 13 measurements from the free viewing test, 16 measurements from the smooth pursuit test, and 6 measurements from the fixation stability test. We chose the examinations of eye movements used in the previously published reports (18, 36, 37). Examples of eye movement examinations are shown in Figure 1. Each paradigm is discussed in detail below.

The free-viewing test was performed using images from five categories that involved buildings, everyday items, foods, fractal patterns, and noise (four images for each category). The subjects were instructed to freely view the presented image for 8 s (Figure 1A). We measured the number and the median duration of the fixations, the number of saccades, the median durations, the amplitude, the mean and the peak velocity of saccades, the scanpath length, and the fixation density (38). The medians over each image were calculated for each eye movement measurement. In addition, we examined the main sequence relation of the saccades of individual subjects (for details, see Supplementary Methods).

In the smooth pursuit eye movement test, subjects were required to track a moving target for 20 s. The target moved horizontally and vertically with a Lissajous trajectory (Figure 1B) and the trial was repeated twice. We measured the number and the median duration of fixations, along with the number, median duration, amplitude, mean, and peak velocities of saccades. In addition, we measured the position gain, the velocity gain, the common logarithm of the signal-to-noise ratio (SNR), and the root mean square error (RMSE) for the horizontal and vertical eye movements separately in each trial.

For the fixation stability test, subjects were required to maintain their gaze on a fixed target presented at the center of the monitor (Figure 1C). A few seconds (1–2 s randomly) after the central fixed target was continually presented, a distracter stimulus appeared at 3° right or left of the central fixed target and presented for 5 s. We measured the number of fixations, the median duration of fixations, the number of saccades, the number of microsaccades, and the scanpath length for each trial. The trial was repeated four times, and we calculated the mean of each eye movement measure over all the trials.

Because some eye movement measurements are influenced by optical devices (39), we divided subjects into naked eye, glasses, and soft contact lens groups and normalized the measurements. Z-scores are dimensionless mathematical tools that allow for the mean-normalization of results within groups. Z-scores are standardized scores (by the group mean and group standard deviation) and no normal assumption is made. They indicate how many standard deviations (σ) an observation (*X*) is above or below the mean of a control group (μ).

$$\begin{array}{l} z\;=\frac{X-\mu }{\sigma }, \end{array}$$

where *X* represents individual data for the observed measurement and μ and σ represent the mean and the standard deviation for the control group, respectively.

### Statistical Analysis

All statistical analyses were performed using SPSS 26.0 (IBM Corp., Armonk, NY, USA). Group comparisons of demographic variables were performed using a two-tailed *t*-test or a χ^2^-test when appropriate.

Because we previously reported that age should be considered when investigating eye movements (40) and other studies also indicated significant effects of age on saccadic eye movements and smooth pursuit eye movements (41, 42), we performed analyses of covariance (ANCOVAs) with the group factor as an independent variable and age as a covariate for z score of each eye measurement. We performed a correction with a false discovery rate (FDR) of 0.05 (Benjamini–Hochberg procedure) for each measurement, considering the nature of multiple comparisons. When significant group-by-age interactions were observed, to evaluate the age effect, we divided MDD and HCs into two cohorts (younger and older cohorts) stratified by the median age of MDD, 48 years [HC group: younger cohort (*N* = 314), older cohort (*N* = 86); MDD group: younger cohort (*N* = 19), older cohort (*N* = 18)]. Then, we examined the simple main effects of group in younger and older cohorts using a general linear model.

For associations between eye movement measurements and demographics, we conducted multiple regression analyses using the stepwise method with eye movement measurements, which showed a significant difference between groups, where eye movement measurements were dependent variables, and age, medication (CPZ, DZP, and IMP equivalents), and HAM-D scores were independent variables. The significance level was set at *p* < 0.05.

A linear discriminant analysis was performed using statistically significant measurements as independent valuables between groups. After the discriminant analysis, the discriminant score was calculated. Optimal sensitivity and specificity of the discriminant score to differentiate between MDD and HCs were determined *via* receiver operating characteristic (ROC) curve analysis using a non-parametric approach. We calculated the Youden index for each cutoff value [(sensitivity + specificity) – 1] to find the cutoff values that maximized the discriminating power.

In addition, 37 HCs were randomly selected to match ages between groups by a technician who was unrelated to this study, and we performed *t*-tests to evaluate age-matched group differences in eye movement measurements. A linear discriminant analysis was also performed.

## Results

### Demographics and Eye Movement Measurements

The demographics of both groups are shown in Table 2. There was no difference in the sex ratio between the two groups (*p* = 0.85) or years of education (*p* = 0.41); however, age was significantly different (*p* = 8.6 × 10^−9^).

Overall, 35 eye movement measurements were obtained in this study. In the HC group, 189 subjects were naked eyes, 124 wore glasses, and 87 had soft contact lenses, whereas 20, 14, and 3 were naked eyes, wore glasses, or had soft contact lenses in the MDD group, respectively (Supplementary Table 1).

### Differences Between Groups

Table 3 shows the mean ± SD of 35 measurements for both groups. In the free-viewing test, there was a significant group difference in scanpath length [*F*_(1, 433)_ = 8.3, *p* = 4.2 × 10^−3^] but the other measurements were not significantly different (0.11 < *p* < 0.94). In the smooth pursuit test, there were significant group differences in duration of saccades [*F*_(1, 433)_ = 8.5, *p* = 3.7 × 10^−3^] and peak saccade velocity [*F*_(1, 433)_ = 8.4, *p* = 3.9 × 10^−3^] but the other measurements were not significantly different (0.02 < *p* < 0.95). There were no significant group differences in the fixation stability test (0.48 < *p* < 0.89).

**Table 3:** Results of ANCOVA.

	**HCs (** ***n*** **=** **400)**		**MDD (** ***n*** **=** **37)**			**Age-by-group interaction**		**Effect of group**	
	**Median**	**SD**	**Median**	**SD**	***df***	***F*-value**	***p*-value**	***F*-value**	***p*-value**
**Free-viewing test**									
Number of fixations	23.00	3.32	21.50	4.93	1,433	5.07	2.5 × 10^−2^^*^	N/A	N/A
Duration of fixation	254.50	46.30	267.00	86.41	1,433	5.51	1.9 × 10^−2^^*^	N/A	N/A
Number of saccades	21.00	3.80	20.00	5.33	1,433	7.19	7.6 × 10^−3^^*^	N/A	N/A
Duration of saccades	42.38	5.59	42.50	6.65	1,433	0.10	0.75	0.91	0.34
Saccade amplitude	4.00	1.17	3.60	0.99	1,433	0.00	0.99	0.66	0.42
Average saccade velocity	93.94	19.25	81.99	17.50	1,433	0.12	0.73	2.51	0.11
Peak saccade velocity	185.10	44.55	181.81	43.85	1,433	0.73	0.39	0.01	0.91
Scanpath length	110.70	28.81	92.56	34.98	1,433	2.19	0.14	8.30	4.2 × 10^−3^^**^
Fixation density	0.88	0.39	0.92	0.51	1,433	9.58	2.1 × 10^−3^^*^	N/A	N/A
Main sequence *v_*max*_*	436.81	119.83	419.68	107.90	1,433	0.39	0.53	0.41	0.52
Main sequence *s*	9.29	4.40	7.63	3.95	1,433	0.00	0.95	0.13	0.72
Main sequence *v_0_*	33.93	9.88	32.60	10.27	1,433	0.57	0.45	0.01	0.94
Number of blinks	1.00	1.49	1.00	1.31	1,433	3.64	0.06	0.45	0.50
**Smooth pursuit test**									
Horizontal SNR	2.03	0.16	2.02	0.18	1,433	2.74	0.10	0.00	0.95
Horizontal position gain	1.01	0.03	1.00	0.03	1,433	0.93	0.33	0.34	0.56
Horizontal RMSE	8.35	4.18	8.78	4.11	1,433	2.30	0.13	0.39	0.53
Vertical SNR	1.84	0.20	1.80	0.20	1,433	1.22	0.27	0.27	0.60
Vertical position gain	0.96	0.07	0.95	0.09	1,433	0.67	0.41	1.18	0.28
Vertical RMSE	14.03	7.94	13.83	9.69	1,433	1.56	0.21	0.15	0.70
Number of fixations	58.25	13.77	60.00	13.99	1,433	0.17	0.68	1.00	0.32
Duration of fixations	259.88	69.00	250.75	79.92	1,433	1.29	0.26	4.27	0.04
Number of saccades	56.00	15.71	59.50	13.85	1,433	0.16	0.69	0.73	0.39
Duration of saccades	30.13	5.63	34.50	9.41	1,433	3.62	0.06	8.54	3.7 × 10^−3^^**^
Saccade amplitude	1.94	0.62	2.38	0.71	1,433	3.22	0.07	5.40	0.02
Average saccade velocity	65.13	11.71	70.80	9.85	1,433	0.00	0.96	1.42	0.23
Peak saccade velocity	98.05	38.71	136.46	46.52	1,433	2.32	0.13	8.41	3.9 × 10^−3^^**^
Horizontal velocity gain	0.85	0.11	0.77	0.14	1,433	0.85	0.36	4.60	0.03
Vertical velocity gain	0.76	0.13	0.70	0.16	1,433	0.59	0.44	1.43	0.23
Number of blinks	1.00	4.00	1.00	2.57	1,433	0.10	0.76	0.24	0.62
**Fixation stability test**									
Number of fixations	3.00	2.46	3.25	2.71	1,433	0.25	0.62	0.10	0.75
Duration of fixation	2057.19	1492.87	1422.50	1558.35	1,433	0.21	0.64	0.50	0.48
Number of saccades	1.50	2.30	2.00	2.53	1,433	0.91	0.34	0.04	0.85
Scanpath length	1.13	2.21	1.36	2.43	1,433	0.23	0.63	0.02	0.89
Number of microsaccades	6.25	3.51	6.50	4.17	1,433	0.46	0.50	0.13	0.72
Number of blinks	0.00	0.97	0.00	0.70	1,433	2.68	0.10	0.04	0.84

*RMSE, Root mean square error; SNR, signal-to-noise ratio; N/A, not applicable*.

* *p < 0.05*.

** *Represents significant after false discovery rate correction*.

### Differences in Younger and Older Cohorts

**Figure 2:**
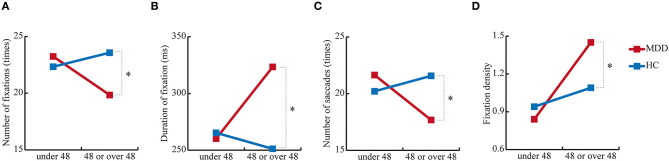
Simple main effects of group. **(A)** Number of fixations, **(B)** duration of fixation, **(C)** number of saccades, and **(D)** fixation density in the free-viewing test. *p < 0.01.

In 4 out of 35 measurements, there were significant interactions between age and group [*F*_(1, 433)_ = 5.1, *p* = 2.5 × 10^−2^) for number of fixations; *F*_(1, 433)_ = 5.5, *p* = 1.9 × 10^−2^ for duration of fixation; *F*_(1, 433)_ = 7.2, *p* = 7.6 × 10^−3^ for number of saccades; and *F*_(1, 433)_ = 9.6, *p* = 2.1 × 10^−3^ for fixation density) (Table 3). For the number of fixations, the group effect was significant in the older cohort [*F*_(1, 433)_ = 17.8, *p* = 2.9 × 10^−5^] but not in the younger cohort [*F*_(1, 433)_ = 1.3, *p* = 0.26]. For the duration of fixation, the group effect was also significant in the older cohort [*F*_(1, 433)_ = 30.7, *p* = 5.3 × 10^−8^] but not in the younger cohort [*F*_(1, 433)_ = 0.19, *p* = 0.67]. For the number of saccades, the group effect was significant in the older cohort [*F*_(1, 433)_ = 15.0, *p* = 1.2 × 10^−4^] but not in the younger cohort [*F*_(1, 433)_ = 2.4, *p* = 0.12]. Finally, for the fixation density, the group effect was significant in the older cohort [*F*_(1, 433)_ = 12.4, *p* = 4.8 × 10^−4^] but not the younger cohort [*F*_(1, 433)_ = 1.3, *p* = 0.26], which suggests altered aging effects in MDD (Figure 2).

### Correlations Between Eye Movement Measurements and Demographics

No correlations were statistically significant with scanpath length in the free-viewing test. The duration of saccades and peak saccade velocity in the smooth pursuit test had no correlations other than age (*R* = 0.44, *p* = 0.02, *R* = 0.47, *p* = 0.01, respectively).

### Discriminant Analysis and ROC Analysis

**Figure 3:**
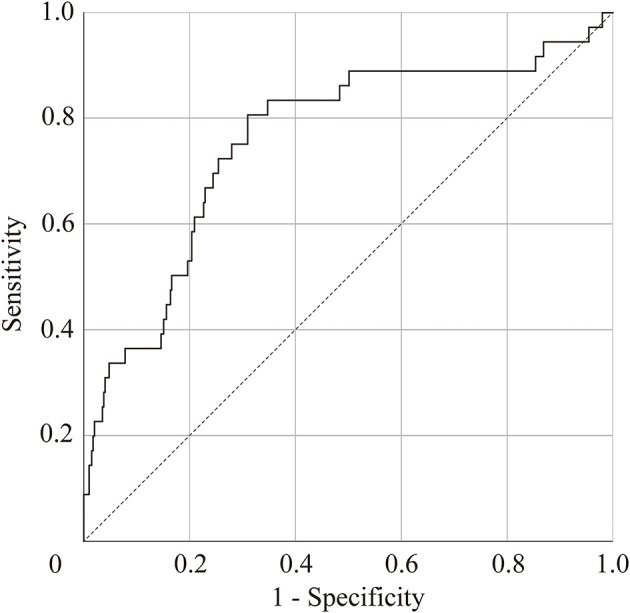
Receiver-operator curve (ROC) to predict patients with major depressive disorder. Area under the ROC = 0.76 (standard error = 0.05, *p* = 1.2 × 10^−7^, 95% confidence interval, 0.67–0.85).

Significant group differences were observed in scanpath length in the free-viewing test, and duration of saccades and peak saccade velocity in the smooth pursuit test. We selected scanpath length of the free-viewing test and peak saccade velocity of the smooth pursuit test for the discriminant analysis using z score for each parameter, because these values were statistically significant between groups and were obtained by independent tests. According to the linear discriminant analysis, we correctly classified 72.1% of the subjects using the resubstitution method and correctly classified 71.9% of the subjects using the leave-one-out cross-validation method. The discriminant score was calculated using the following formula: discriminant score = – 0.52 × (z score of scanpath length) + 0.83 × (z score of peak saccade velocity). Figure 3 shows the ROC curve of the discriminant score between the MDD and HC groups. The area under the curve (AUC) of the ROC analysis in MDD vs. HCs was 0.76 (standard error = 0.05, *p* = 1.2 × 10^−7^, 95% CI = 0.67 – 0.85), indicating that the discriminant score for the scanpath length in the free-viewing test and the peak saccade velocity in the smooth pursuit test could be used to differentiate between MDD and HC subjects with moderate accuracy. The Youden index indicated a favorable cutoff point of 0.39, which resulted in 81% sensitivity and 69% specificity.

### Age-Matched Group Analysis

The results of the *t*-test are shown in Supplementary Table 2. These were: the scanpath length for the free viewing test (raw *p* = 0.01, *d* = 0.6); the duration of saccades and peak saccade velocity for the smooth pursuit test (raw *p* = 3.9 × 10^−3^, *d* = −0.7; raw *p* = 1.5 × 10^−3^, *d* = −0.8, respectively), but none of them were significant after FDR correction. According to the linear discriminant analysis, we correctly classified 75.7% of the subjects using the resubstitution method and correctly classified 73.0% of the subjects using the leave-one-out cross-validation method. The discriminant score was calculated using the following formula: discriminant score = – 0.63 × (scanpath length) + 0.80 × (peak saccade velocity). Supplementary Figure 1 shows the ROC curve of the discriminant score between the MDD and HC groups in age-matched group analysis. AUC of the ROC analysis in MDD vs. HCs was 0.77 (standard error = 0.06, *p* = 6.5 × 10^−5^, 95% CI = 0.66 – 0.88). The Youden index indicated a favorable cutoff point of 0.03, which resulted in 73% sensitivity and 81% specificity.

## Discussion

The present study investigated eye movement measurements of MDD using free-viewing, fixation stability, and smooth pursuit tests. The results are as follows: (1) MDD showed a significantly shortened scanpath length in the free-viewing test and a longer duration of saccades and higher peak saccade velocity in the smooth pursuit test compared with HCs; (2) altered aging effects of MDD were observed for the number of fixations, duration of fixation, number of saccades, and fixation density; and (3) the AUC of the ROC analysis was 0.76 (standard error = 0.05, *p* = 1.2 × 10^−7^, 95% CI = 0.67 – 0.85).

The current findings are partially consistent with a previous study (31) that reported MDD patients exhibited fewer saccades and a shortened scanpath length in the free-viewing task compared with HCs. In addition, they found that MDD patients had a significantly shorter gaze time and more saccades in the fixation stability test. However, in the present study, there were no significant differences in the fixation stability test between MDD and HC subjects. These differences between studies might be because the previous study by Li et al. (31) did not compensate for multiple tests, and thus their results should be interpreted carefully. Hsu et al. (43) investigated temporal preparation in MDD using a saccadic eye movement task where subjects were required to make a saccade between a central and an eccentric visual target. Patients with MDD showed a larger number of saccades initiated prior to the appearance of the expected stimulus, indicating reduced temporal preparation in MDD. In addition, the authors reported that oculomotor impulsivity interacted with temporal preparation. In our study, there were no significant differences in the duration of fixation and the number of saccades in the fixation stability test between groups, suggesting that oculomotor impulsivity was not observed in MDD. However, differences in experimental design may account for the reported discrepancies.

Our previous study (20) reported that during the free-viewing test, patients with SZ also had a significantly shortened scanpath length compared with HCs. Based on this result, it appears that the decline in the scanpath length in MDD may be less severe than that in SZ. Egaña et al. (44) reported significant shortened scanpath in SZ compared with HCs was resulted from the increasing occurrence of undetected microsaccades. Our future study will examine this issue for shortened scanpath length in MDD. In the smooth pursuit test, there were significant declines in horizontal and vertical position gains in SZ, but there were no significant changes in position gain in MDD. However, there were significant differences in the duration of saccades and peak saccade velocity, which may indicate that patients with MDD compensate with position error by catch-up saccade. It will be necessary to clarify the commonality and heterogeneity of eye movement parameters in psychiatric disorders in future studies.

Altered aging effects of MDD including the number of fixations, duration of fixation, number of saccades, and fixation density are also of particular interest. Indeed, accelerated brain aging was reported in MDD patients (45). In terms of symptoms, Dunlop et al. (46) suggested that accelerated aging was associated with greater impulsivity and depression severity. A genetic study by Han et al. reported that patients with MDD had a degree of epigenetic and methylation change that was reflective of an older age. In particular, they suggested that MDD patients were 8 months biologically older than people without MDD (47). The current finding on different patterns of aging effects between MDD and HCs suggests that MDD patients may show different age-related changes from HCs, which could relate to disease characteristics. We hope to confirm these findings in a larger cohort in the future.

The results of discriminant analysis and ROC analysis suggest that the combination of scanpath length by free-viewing test and peak saccade velocity by smooth pursuit test are potential biomarkers to differentiate between MDD and HCs. In the present study, we also performed age-matched group analysis and found no significant differences after FDR correction in the eye movement variables that were significantly different in the main ANCOVA. However, in terms of effect sizes comparing MDD and HCs, the impacts of eye movements seem to be more significant compared to other indices. For example, in a structural brain imaging study with mega-analysis methods (48), patients with MDD had a significantly thinner cortical gray matter in the orbitofrontal cortex, anterior cingulate gyrus, posterior cingulate gyrus, insula, and temporal lobes compared to HCs (Cohen's *d* effect size: −0.10 to −0.14), whereas the effect sizes of our present study were 0.60–0.77. In addition, the study using MRI as a biomarker reported that patients with MDD were distinguished from HCs with a sensitivity of 77% and specificity of 78% (49), which was comparable to the present results. Therefore, it may be reasonable to select variables of eye movements as potential biomarkers. It will be important to utilize the same measurements for the differentiation of other psychiatric disorders. Further study of the eye movement measurements in psychiatric disorders other than SZ and MDD, such as bipolar disorder, anxiety disorder, obsessive-compulsive disorder, and autism spectrum disorder may identify eye movement-related biological differences across psychiatric disorders. In future clinical applications, it may be necessary to narrow down the parameters to be used.

Several limitations of this study must be considered. First, differences in group age might have influenced the discrimination analysis because we used the peak saccade velocity in the smooth pursuit test as an independent variable, which is associated with age. To obtain more accurate results, demographically-matched groups should be used in future analyses. Second, patients with MDD had various disease status including mild-to-moderate severity and remission with medication. Furthermore, the sample size was relatively small. Thus, larger numbers of samples with all types of depressive states ranging from mild to severe will be required to determine whether the abnormalities are traits or a state of the disorder. As shown in age-matched group analyses, although the effect sizes were large, significant differences would only be found with larger sample sizes in an exploratory study. Therefore, we need to confirm further our results in another extensive age-matched data set as a confirmatory study. Third, the current study cannot answer the question of whether the current findings are specific to MDD or not. It will thus be important to investigate other psychiatric disorders such as bipolar disorder. Our future study will perform direct comparisons among disorders, including schizophrenia, with larger sample size. Finally, the neural basis of these age-related eye-movement abnormal changes remains poorly understood. Thus, to determine the neural basis of these age-related eye-movement abnormalities, functional neuroimaging studies including functional magnetic resonance imaging, electroencephalography, or magnetoencephalography should be combined during the eye-movement tasks.

## Conclusion

In the current study, MDD patients had a significantly shortened scanpath length in the free-viewing test and a longer duration of saccades and higher peak saccade velocity in the smooth pursuit test compared with HCs. In addition, altered aging effects of MDD were detected for the number of fixations, duration of fixation, number of saccades, and fixation density. The discriminant score calculated by the scanpath length in the free-viewing test and peak saccade velocity in the smooth pursuit test might be used to differentiate between MDD and HCs with moderate accuracy. These results suggest that detailed eye movement tests can assist in differentiating MDD from HCs, especially in older subjects.

## Data Availability Statement

The raw data supporting the conclusions of this article will be made available by the authors, without undue reservation.

## Ethics Statement

The studies involving human participants were reviewed and approved by Research Ethical Committees of Kyushu University, Osaka University, The University of Tokyo, Nagoya University, and Nara Medical University. The patients/participants provided their written informed consent to participate in this study.

## Author Contributions

JT, KMi, RH, YH, and TO designed the study. JT, KMi, KMo, MF, HY, YY, NK, ES, KO, and TS collected and analyzed the data. JT prepared the first draft of the manuscript and created the figures. KMi, YH, RH, and TO assisted with data analyses and supervised the research. KMi, KMo, MF, HY, YY, NK, ES, KO, TS, TN, KK, RH, and TO edited the manuscript. All authors contributed to and have approved the final manuscript.

## Conflict of Interest

The authors declare that the research was conducted in the absence of any commercial or financial relationships that could be construed as a potential conflict of interest.

## Publisher's Note

All claims expressed in this article are solely those of the authors and do not necessarily represent those of their affiliated organizations, or those of the publisher, the editors and the reviewers. Any product that may be evaluated in this article, or claim that may be made by its manufacturer, is not guaranteed or endorsed by the publisher.

## Supplementary Material

Supplementary Figure 1 Receiver-operator curve (ROC) to predict patients with major depressive disorder. Area under the ROC = 0.77 (standard error = 0.06, *p* = 6.5 × 10^−5^, 95% confidence interval, 0.66–0.88).

Supplementary Table 1 Eye movement measurements of HCs and MDD subjects.

Supplementary Table 2 Results of *t*-tests between age-matched groups.
