# Supplementary material for: Eye Movement Abnormalities in Major Depressive Disorder
Source: Front Psychiatry. 2021 Aug 10;12:673443. doi: 10.3389/fpsyt.2021.673443 (PMC8382962; doi:10.3389/fpsyt.2021.673443)
Supplement: Supplementary file 1 [file Data_Sheet_1.docx]

**Eye movement recordings and processing of the data**

The eye position data (in degrees) were smoothed with a digital FIR filter (-3 dB at 30 Hz), and the eye velocity traces were derived from the two-point forward difference. The eye acceleration profiles were derived from the two-point forward difference of the eye velocity traces. Then, the eye movement records were segmented into the blink, saccade and fixation periods. First, we identified the periods of blink during which data were missing and the periods of high-speed deflections of the eye positions as candidates of the saccade periods (eye velocity >35°/s, eye acceleration >5,000°/s^2^). The periods of high-speed deflections could involve artifacts associated with blinks that were distinguished by the following criteria. The high-speed deflections that followed or were followed by blink periods (within 50 ms) were likely to be artifacts associated with eyelid closures during the blinks; these periods were incorporated into the corresponding blink periods. The high-speed deflections that were accompanied by changes in the pupil area (>25 units/samples) were likely to be artifacts associated with blinks with incomplete eyelid closures, which were identified as the blink periods. Because the saccade criteria previously described were set to be low to detect small saccades, the periods that involved small positional noise could sometimes be captured. To avoid inclusions of these periods into the saccade list, the periods with too small deflections (0.1 deg), too short/long duration (<10 ms or >300 ms) or too high accelerations (±0.6×10^5^) were eliminated from the list of candidates, and the other high-speed deflection periods were identified as the saccade periods. The remaining periods were identified as the fixation periods if the periods were longer than 20 ms; if the periods were shorter than 20 ms, they were considered as the unidentified periods. The saccades toward/from outside of the screen and the fixations outside the screen were marked and excluded when the eye movement variables were calculated.

**Eye movement tests and variables extracted**

We administered three eye movement examinations and obtained 35 eye movement variables.

In the free viewing test, we obtained 13 variables. The scanpath length is the sum of the distances (degree) between the average positions of neighboring fixations over time. The fixation density is a measure of skewness represented as the parameter b of Gamma distribution fitted to the distributions of Voronoi cell sizes (Over et al., 2006). We also examined the main sequence relationship of the saccades of individual subjects by fitting the function V= *v_max_*×{1-exp (-*s*×A)}+ *v_0_* to the amplitude (A) and peak eye velocity (V) of the saccades obtained from all trials, where *v_max_*, *s* and *v_0_* were optimized.

In the smooth pursuit eye movement test, the subjects were required to track a moving target for twenty seconds. The target moved on Lissajous trajectories at the speed: 0.3 Hz (±18.1°/s) in horizontal and 0.4 Hz (±24.2°/s) in vertical axes. The trial was repeated twice. We obtained 16 variables. We applied Fast Fourier Transform (FFT) to the eye position data using an 8192-point Hamming window. The SNR was defined as the power of ±0.1-Hz range of the target's frequency (the signal) divided by the average power over 0.8-8 Hz (noise) (Muir et al., 1992). The position gain was defined as the power of the eye movement signal relative to the target motion. For the velocity gain, we applied FFT to the eye velocity data after removing and linearly interpolating the interval during the blinks and saccades. The power of ±0.1-Hz range of the target's frequency relative to the target motion was calculated as the velocity gain. Fifty samples were obtained by randomly selecting the start time of the window and then calculating the median values for each variable.

**References**

Over EA, Hooge IT, Erkelens CJ. A quantitative measure for the uniformity of fixation density: The Voronoi method. *Behav. Res. Methods* (2006) 38, 251–261.

Muir, W.J., St Clair, D.M., Blackwood, D.H., Roxburgh, H.M., Marshall, I. Eye-tracking dysfunction in the affective psychoses and schizophrenia. *Psychol Med*. (1992) 22, 573-580.
