# Supplementary figures and images for: Eye Movement Abnormalities in Major Depressive Disorder
Source: Front Psychiatry. 2021 Aug 10;12:673443. doi: 10.3389/fpsyt.2021.673443 (PMC8382962; doi:10.3389/fpsyt.2021.673443)

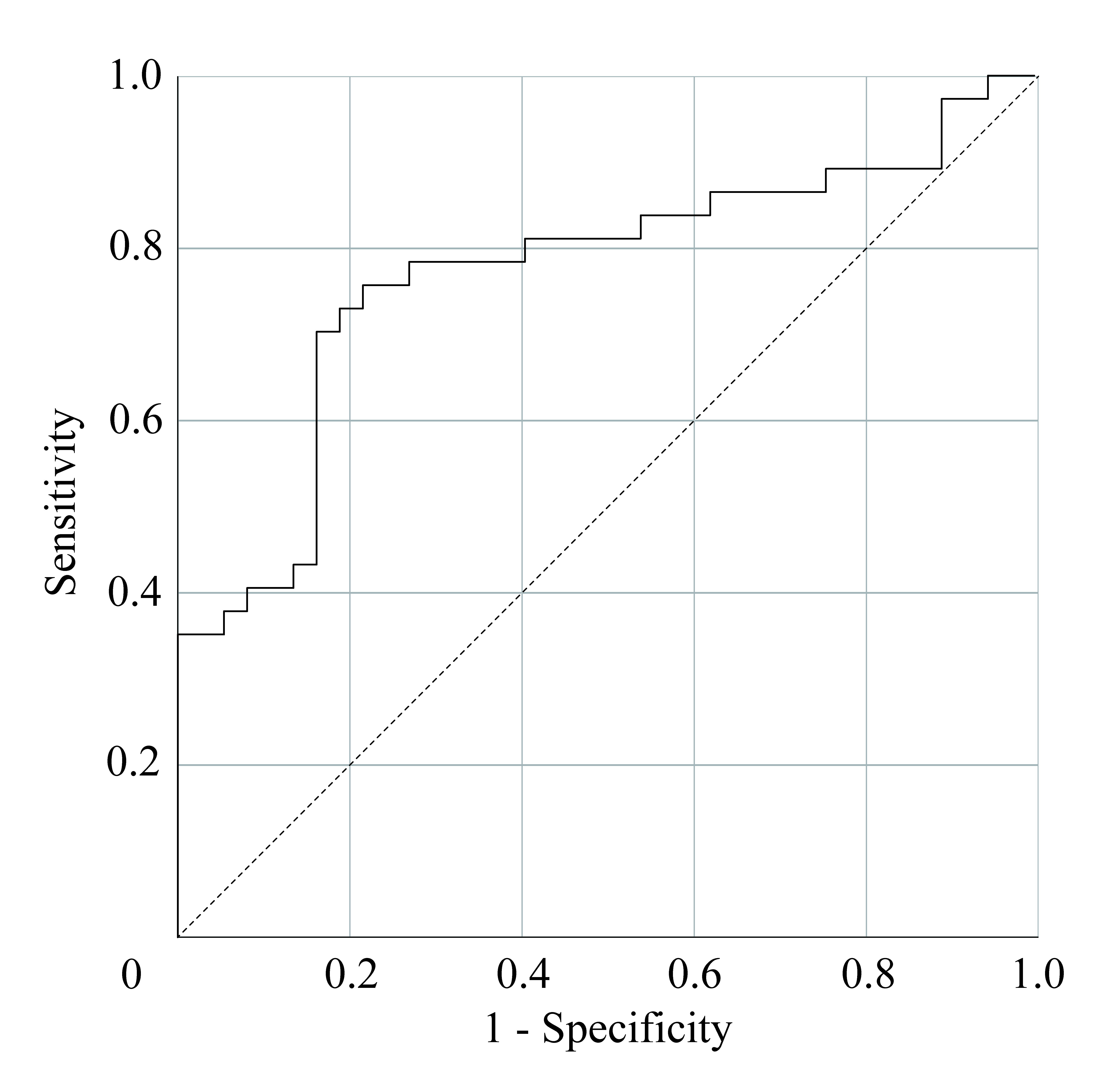

Supplement: Supplementary Figure 1 — Receiver-operator curve (ROC) to predict patients with major depressive disorder. Area under the ROC = 0.77 (standard error = 0.06, p = 6.5 × 10^−5^, 95% confidence interval, 0.66–0.88). [file Image_1.JPEG]
